# Genetic Dissection of Seed Dormancy in Rice (*Oryza sativa* L.) by Using Two Mapping Populations Derived from Common Parents

**DOI:** 10.1186/s12284-020-00413-4

**Published:** 2020-08-05

**Authors:** Chaopu Zhang, Zhiyang Yuan, Yuntong Wang, Wenqiang Sun, Xinxin Tang, Yongjian Sun, Sibin Yu

**Affiliations:** National Key Laboratory of Crop Genetic Improvement, College of Plant Science and Technology, Huazhong Agricultural University, Wuhan, 430000 China

**Keywords:** Rice, Seed dormancy, Chromosome segment substitution lines, Backcross inbred lines, QTL

## Abstract

**Background:**

Seed dormancy, a quality characteristic that plays a role in seed germination, seedling establishment and grain yield, is affected by multiple genes and environmental factors. The genetic and molecular mechanisms underlying seed dormancy in rice remain largely unknown.

**Results:**

Quantitative trait loci (QTLs) for seed dormancy were identified in two different mapping populations, a chromosome segment substitution line (CSSL) and backcross inbred line (BIL) population, both derived from the same parents Nipponbare, a *japonica* cultivar with seed dormancy, and 9311, an *indica* cultivar lacking seed dormancy. A total of 12 and 27 QTL regions for seed dormancy were detected in the CSSLs and BILs, respectively. Among these regions, four major loci (*qSD3.1*, *qSD3.2*, *qSD5.2* and *qSD11.2*) were commonly identified for multiple germination parameters associated with seed dormancy in both populations, with Nipponbare alleles delaying the seed germination percentage and decreasing germination uniformity. Two loci (*qSD3.1* and *qSD3.2*) were individually validated in the near-isogenic lines containing the QTL of interest. The effect of *qSD3.2* was further confirmed in a CSSL-derived F_2_ population. Furthermore, both *qSD3.1* and *qSD3.2* were sensitive to abscisic acid and exhibited a significant epistatic interaction to increase seed dormancy.

**Conclusions:**

Our results indicate that the integration of the developed CSSLs and BILs with high-density markers can provide a powerful tool for dissecting the genetic basis of seed dormancy in rice. Our findings regarding the major loci and their interactions with several promising candidate genes that are induced by abscisic acid and specifically expressed in the seeds will facilitate further gene discovery and a better understanding of the genetic and molecular mechanisms of seed dormancy for improving seed quality in rice breeding programs.

## Background

Seed dormancy is defined as the inability of seeds to germinate under favorable conditions, and the loss of dormancy is assumed to be a typical domestication trait in crops (Bentsink et al. 2006; Wang et al. 2018). It is also an important agronomic trait related to seed quality and grain yield. High seed dormancy causes nonuniformity of germination and poor seedling establishment (Anderson et al. 1993). Low seed dormancy can ensure the uniformity of germination but is likely to result in preharvest sprouting during the maturity stage, causing a serious reduction in the production and grain quality of cereal crops (Fang et al. 2008; Sugimoto et al. 2010; Marzougui et al. 2012). Therefore, appropriate seed dormancy is a desirable characteristic for most crops, and a better understanding of the genetic and molecular mechanisms involved is essential for improving seed quality in plant breeding programs.

Seed dormancy is a complex trait affected by multiple genes and environmental factors (Graeber et al. 2014; Lu et al. 2018) and by multiple hormones, such as abscisic acid (ABA) and gibberellins (GA), which antagonistically regulate seed dormancy (Finkelstein et al. 2008; Shu et al. 2016; Née et al. 2017a, 2017b). In rice (*Oryza sativa* L.), large variations in seed dormancy are observed between the two cultivated subspecies *japonica* and *indica*. More than 150 quantitative trait loci (QTLs) associated with seed dormancy have been identified in various biparental mapping populations (Lin et al. 1998; Cai and Morishima 2000; Wan et al. 2006; Gu et al. 2010; Marzougui et al. 2012; Mizuno et al. 2018; Nguyen et al. 2019) (https://archive.gramene.org/qtl/). In addition, several loci affecting seed dormancy have been detected through genome-wide association mapping in rice (Magwa et al. 2016; Lu et al. 2018). Although a large number of QTLs for seed dormancy have been identified in rice, only a few of them have been subjected to map-based cloning to identify the underlying genes. For instance, *seed dormancy 4* (*Sdr4*) on chromosome 7 (23.79 Mb), which encodes a protein of unknown function, was reported as the first cloned QTL in rice and is positively regulated by the seed maturation-related gene *OsVP1* (Sugimoto et al. 2010). *SD7–1/Rc*, also on chromosome 7 (6.06 Mb), which has been demonstrated to influence red pericarp color and seed dormancy, encodes a basic helix-loop helix transcription factor involved in ABA synthesis (Gu et al. 2011). *Seed dormancy1–2* (*qSD1–2*), also known as the green revolution gene *semidwarf-1* (*sd-1*), is located on chromosome 1 (38.38 Mb) and induces seed endosperm-imposed dormancy (Ye et al. 2015). Recently, *OsG*, on chromosome 3 (0.013 Mb) has been shown to encode an amino-terminal protease protein in rice, and its orthologous stay-green genes *G* in soybean (*Glycine max* L. Merr.) and *AtG* in *Arabidopsis* (*Arabidopsis thaliana* (L.) Heynh.) have been shown to be responsible for seed dormancy by interacting with nine-cis-epoxycarotenoid dioxygenase 3 and phytoene synthase, which are involved in ABA biosynthesis (Wang et al. 2018). In addition, more than 120 QTLs affecting seed dormancy have been detected in other model plants and crops (https://archive.gramene.org/qtl/), such as *Arabidopsis* (Bentsink et al. 2006), wheat (*Triticum aestivum* L.) (Anderson et al. 1993), oilseed rape (*Brassica napus* L.) (Schatzki et al. 2013), barley (*Hordeum vulgare* L.) (Nagel et al. 2019) and sorghum (*Sorghum bicolor* L.) (Cantoro et al. 2016). In addition, many genes related to seed dormancy, such as *DELAY OF GERMINATION 1* (*DOG1*), have been identified and intensively characterized in *Arabidopsis* (Shu et al. 2016). *DOG1* encodes a protein of unknown function and is regulated at the transcriptional and posttranscriptional levels in *Arabidopsis* (Bentsink et al. 2006; Müller et al. 2012; Chen and Penfield 2018). Overall, these data indicate that the genetic and molecular mechanisms underlying seed dormancy are very complex in *Arabidopsis* and other plant species. Therefore, it is critical to identify the QTLs related to seed dormancy to dissect the molecular basis of this complex trait.

The objective of the present study was to identify QTLs for seed dormancy and elucidate the genetic basis of seed dormancy in rice. Initially, we evaluated both a chromosome segment substitution line (CSSL) and backcross inbred line (BIL) population for seed dormancy. Both populations were derived from the same parents, Nipponbare (NIP), a *japonica* cultivar with seed dormancy crossed with 9311, an *indica* cultivar lacking seed dormancy. SNP genotyping identified 12 and 27 QTLs for seed dormancy in CSSL and BIL populations, respectively, with four major QTLs (*qSD3.1*, *qSD3.2*, *qSD5.2* and *qSD11.2*) revealed for multiple germination parameters in both populations. Subsequently, two major QTLs (*qSD3.1* and *qSD3.2*) detected on chromosome 3 were validated using near-isogenic lines (NILs) and segregating populations. Further investigation detected a digenic interaction between *qSD3.1* and *qSD3.2*, which is assumed to be involved in the ABA regulation of seed dormancy. Our results provide new insights into the genetic basis of seed dormancy for the improvement of seed quality by marker-assisted selection breeding in rice.

## Materials and Methods

### Plant Materials

The CSSL and BIL populations were used to dissect the genetic basis of seed dormancy in rice. These two populations were derived using different approaches from the same cross between the two genome-sequenced rice cultivars, NIP and 9311. NIP is classified as subspecies *japonica* and exhibits seed dormancy, whereas 9311, an elite restorer line is classified as subspecies *indica* and lacks seed dormancy. The first population of the CSSLs consisted of 122 lines and was developed from a cross between NIP (as the donor) and 9311 (as the recurrent parent) using a backcross scheme with a marker-assisted selection approach (Additional file 1: Fig. S1). In the CSSLs, each line contained one or a few introduced NIP segments within the 9311 background (Tan et al. 2011). The other population (BILs) comprising 437 lines derived from a NIP × 9311 F_1_ backcrossed to 9311 and advanced to the F_8_ by single-seed descent (Additional file 1: Fig. S1). The genomic DNA from each line of BILs was extracted and subjected to genotyping as described previously (Yuan et al. 2019). To validate the effect of *qSD3.2*, one CSSL (NY38) that carried a 1.6-Mb introduced NIP segment surrounding *qSD3.2* on chromosome 3 and a background NIP segment on chromosome 10 was crossed with 9311 to generate an F_2_ population (hereafter referred to as the NY38-derived population). The other CSSL (NY61) harboring two introduced NIP segments surrounding *qSD3.1* and *qSD3.2,* was used to generate an additional F_2_ population (referred to as the NY61-derived population). Two near-isogenic lines (NILs) containing either *qSD3.1* or *qSD3.2* were obtained during the development of these mapping populations. The NILs and the mapping populations along with the parental lines were grown at the Wuhan Experimental Station of Huazhong Agricultural University, China. Each line was planted in four rows with ten individuals per row with spacing of 16.7 × 26.6 cm. Field management was carried out according to the local standard practices (Tan et al. 2011).

### Seed Dormancy Evaluation

The flowering time was recorded as the time of the appearance of the panicle from the flag leaf sheath. Seeds were harvested from individual plants at 35 days after flowering and then equilibrated for 5–6 days at 15% RH (called freshly harvested seeds) to ensure the uniformity of the seed moisture content (approximately 12%) for all lines. Then, the seeds from ten individuals of each line were pooled, packaged and stored at − 20 °C to maintain their dormant status for germination experiments. Germination testing of every sample was performed with three replicates. Fifty healthy seeds from each sample were placed in a Petri dish (diameter 9 cm) with two sheets of moistened filter paper for the germination test (Gu et al. 2011). All dishes were placed in a growth chamber (Dongnan, Ningbo, China) at 25 °C under a 16 h light/ 8 h dark cycle. The number of germinated seeds was monitored and counted every 24 h for seven consecutive days to construct cumulative germination curves (Yuan et al. 2019). Freshly harvested seeds were treated at 43 °C for 3 days to break seed dormancy as described previously (Li et al. 2011). Germination assays were also conducted for the treated seeds, which were referred to as after-ripened seeds. Germination parameters were calculated from the germination curve using the Germinator package (Joosen et al. 2010). Four parameters were used to assess seed dormancy, including G_3d_, the germination rate at 72 h after imbibition; G_7d_, the maximum germination rate at 168 h after imbibition; T_50_, the germination speed, which is the time required to reach 50% germination of seeds; and AUC, the area under the curve over 168 h after imbibition, which is the integration of the fitted curve between t = 0 and t = 168 that represents germination uniformity.

### SNP Genotyping and Bin-Map Construction

Previously, 165 simple sequence repeat markers were used for the detection of the introduced NIP segments in the NIP × 9311 CSSL population (Tan et al. 2011). However, the small segments in the population might be falsely interpreted because of the low density of traditional markers. To overcome these problems and more precisely confirm the genotypes, the 122 CSSLs were reanalyzed with a RICE6K chip (Yu et al. 2014) and used in this study.

For the 437 BILs, a genotyping-by-sequencing (GBS) strategy with high-throughput genotyping was adopted to confirm the genotypes (Yuan et al. 2019). A total of 49,890 high-quality single-nucleotide polymorphisms (SNPs) were identified after filtering out the low-quality SNPs, followed by Bayesian inference (Yu et al. 2011). The heterozygous genotypes were set as missing data in the BILs, and the lines with > 20% missing data were excluded from the BILs for further analysis. The bin maps were constructed based on the physical locations of recombination breakpoints and SNP genotypes as previously described with minor modification (Huang et al. 2009; Li et al. 2017). Briefly, the genotype of each line was scanned with a sliding window of 15 SNPs and a step size of 1. An “a/b” ratio of 12:3 or higher was recorded as “a”, and a ratio of 3:12 or lower was recorded as “b”. The missing genotypes were coded as “-”. Adjacent windows with the same genotype were combined into a block, and the recombinant breakpoints were assumed to occur at the boundary of adjacent blocks with different genotypes. The interval between two adjacent crossovers in the entire population was defined as a recombination bin.

### QTL Analysis

The QTL analysis of the phenotypic data with bin-maps in the CSSLs and BILs was performed using the linear ridge regression method to reduce the multicollinearity among markers as described previously (Sun et al. 2015). A significance level of *P* < 0.005 was set as the threshold in the CSSLs and BILs to declare the presence of a putative QTL in a given bin. If several adjacent bins showed *P* values lower than the threshold, the QTL was tentatively located in the bin (peak bin) with the lowest *P* value (Sun et al. 2015). The phenotypic variance explained by each QTL was decomposed using the “relaimpo” package of R (“lmg” function). QTL nomenclature followed the principles suggested by a previous report (McCouch 2008). QTL analysis in the F_2_ population was performed using QTL IciMapping V4.1 (Meng et al. 2015). This F_2_ population was genotyped by using eight markers in the introgression region of chromosome 3 (Additional file 2, Table S1). The epistatic interaction of target QTLs genotyped by using MP30026 and C32827 in the segregating population was analyzed by two-way analysis of variance (ANOVA) in R software (http://www.r-project.org/).

Gene annotations for a given peak bin were obtained from the Rice Genome Annotation Project Database (http://rice.plantbiology.msu.edu/). Putative candidate genes were selected after the removal of genes annotated as unknown, transposons/retrotransposons, or hypothetical proteins and genes showing no sequence divergence or nonsynonymous mutation between NIP and 9311 or almost no expression in the seeds. Sequence variations were obtained from RiceVarMap V2.0 (http://ricevarmap.ncpgr.cn/v2/). Gene expression patterns were obtained from the Rice Genome Database (http://rice.plantbiology.msu.edu/ expression.shtml).

## Results

### Seed Dormancy of CSSLs and BILs

**Fig. 1:**
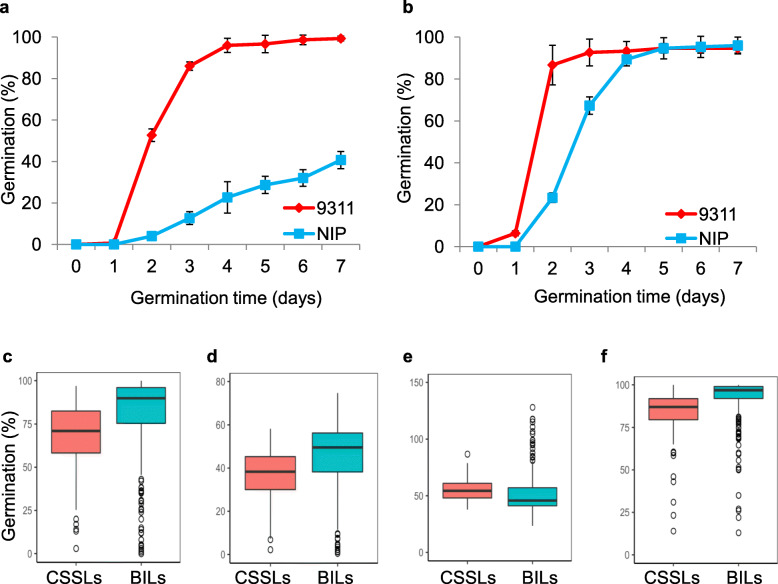
Differences in seed dormancy between Nipponbare (NIP) and 9311 and among chromosome segment substitution lines (CSSLs) and backcross inbred lines (BILs). **a** Germination curve of the freshly harvested seeds of NIP and 9311. **b** Germination curve of NIP and 9311 after seed dormancy was broken. The error bar represents the mean ± SD (*n* = 3). Boxplots of G_3d_ (**c**), AUC (**d**), T_50_ (**e**), and G_7d_ (**f**) in CSSLs and BILs. Box edges indicate the range of the 25th to 75th percentiles, with the median value shown by the bold middle line. Whiskers represent the range of 5% to 95% of the data, and outer dots are outliers. G_3d_, germination rate at 72 h after imbibition; G_7d_, maximum germination rate at 168 h after imbibition; T_50_, germination speed, which is the time to reach 50% germination of seeds; and AUC, the area under the curve up to 168 h after imbibition

Two mapping populations (CSSLs and BILs) were developed from the common parents NIP and 9311 and used to evaluate the genetic basis of seed dormancy in rice (Additional file 1: Fig. S1). The two parents NIP and 9311 exhibited significant differences in seed dormancy as assayed according to four germination parameters. The freshly harvested seeds of NIP showed significantly delayed germination compared with 9311. The germination rate at day 7 after imbibition (G_7d_) in NIP was 40.6%, but that in 9311 was greater than 95% (Fig. 1a). In addition, NIP presented a significantly lower germination rate on day 3 after imbibition (G_3d_), a significantly smaller area under the curve up to 168 h (AUC) and a longer time to achieve 50% germination of seeds (T_50_) than 9311 (Additional file 3: Table S2). The after-ripened seeds of NIP and 9311 showed a similar G_7d_ to 9311, indicating that the seeds of the two parents presented the same high seed viability after dormancy was broken (Fig. 1b). These results indicate that NIP exhibits stronger seed dormancy than 9311.

The CSSLs and BILs showed wide phenotypic variations in four germination parameters (Fig. 1c-f), exhibiting a quantitative trait inheritance pattern. The majority of the lines had seed dormancy similar to 9311 but several lines had significantly higher or lower values than 9311. This suggests these lines with more extreme values may carry the introduced NIP segments containing the loci associated with seed dormancy. Moreover, the BILs exhibited even broader variations in the four parameters than CSSLs (Additional file 3: Table S2), indicating that the BILs present a more complex genetic basis for seed dormancy, which may be caused by genetic interaction effects. Correlation analysis was performed for the four parameters in the CSSLs and BILs. Significant positive correlations were observed among three parameters (Additional file 4: Fig. S2), including G_3d_, G_7d_ and AUC, whereas these three parameters showed significantly negative correlations with T_50_ in both populations.

### Genotyping of CSSLs and BILs and Construction of Bin Maps

Genotyping of the 122 CSSLs by using a RICE6K chip generated a total of 3383 high-quality SNPs, which were evenly distributed on all 12 chromosomes (Additional file 5: Table S3). These SNPs were then used as potential markers for bin-map construction. The identified SNP genotypes revealed that each CSSL carried one or a few NIP segments in the 9311 background, with an average genome coverage of 96.7%. To identify the QTLs for seed dormancy, a bin map based on the recombination breakpoints in the CSSLs was constructed. A total of 387 bins were generated with a median length of 800 kb (Additional file 5: Table S3). For the 437 BILs, a total of 49,890 high-quality SNPs were identified by using a GBS strategy (Additional file 5: Table S3). Thirty-seven lines in the population were excluded due to showing > 20% missing genotype data. Thus, the bin map was generated with 3235 bins in 400 BILs following the procedure described previously (Huang et al. 2009). The bin lengths ranged from 30 kb to 3.0 Mb with an average of 115 kb in the BILs (Additional file 6: Table S4).

### Detection of QTLs for Seed Dormancy in CSSLs

**Fig. 2:**
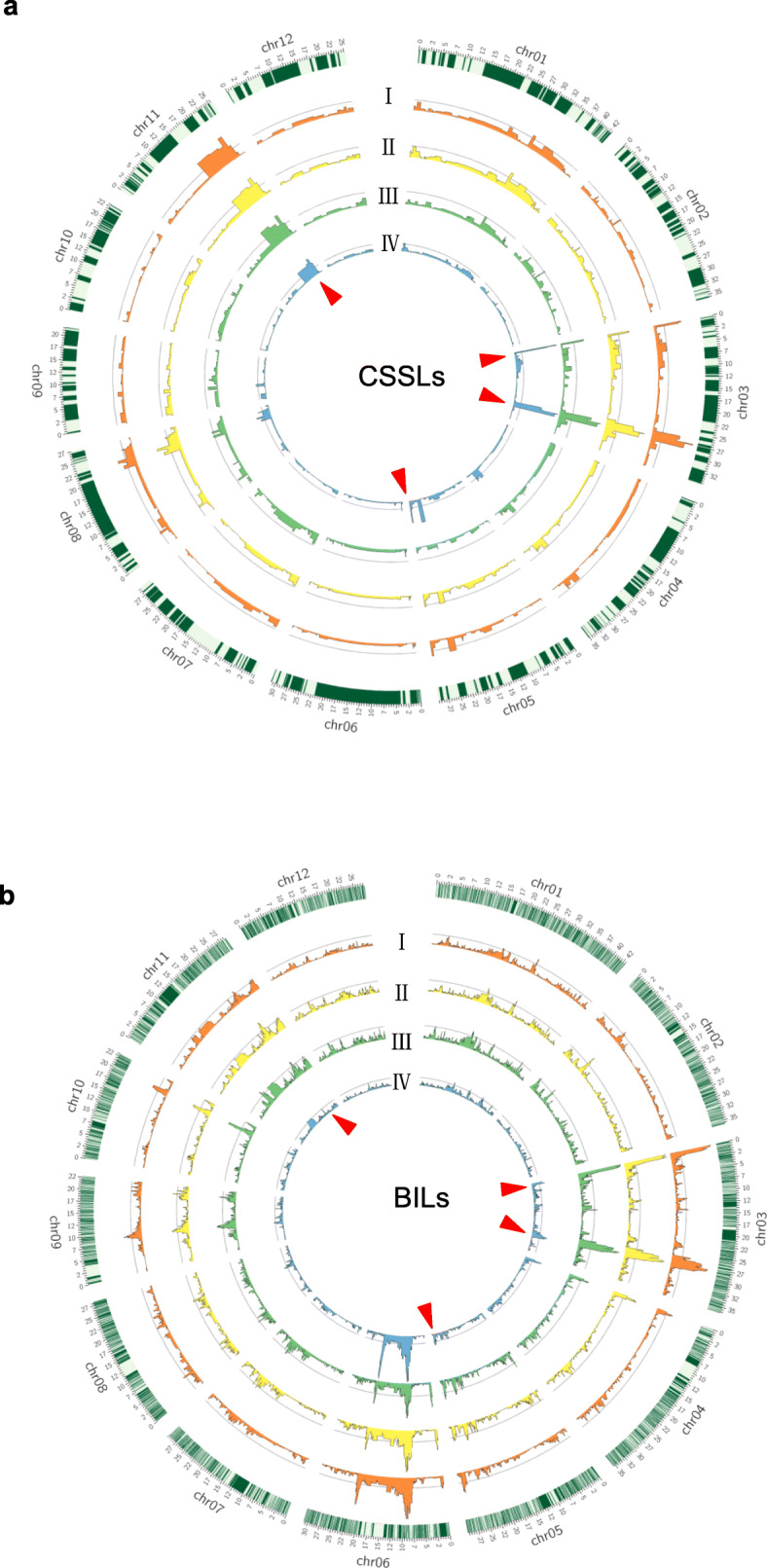
Genome-wide distribution of quantitative trait loci (QTLs) detected for four seed dormancy parameters in the two populations. **a** CSSLs (chromosome segment substitution lines). **b** BILs (backcross inbred lines). Rice chromosomes with bins are indicated in the outer circle. The outer to the inner circles represent G_3d_ (I), AUC (II), T_50_ (III) and G_7d_ (IV), respectively. G_3d_, germination rate at 72 h after imbibition; G_7d_, maximum germination rate at 168 h after imbibition; T_50_, germination speed, which is the time to reach 50% germination of seeds; and AUC, the area under the curve up to 168 h after imbibition. Red arrows represent the major loci associated with seed dormancy in both populations. For each bar diagram, the *x*-axis represents the physical location along each numbered chromosome. The *y*-axis represents the *P* value for the single-nucleotide polymorphism (SNP) association. Dashed lines indicate the declaration thresholds

The linear ridge regression method was used for QTL mapping in the CSSL population to decrease the multicollinearity among markers as described in a previous study (Sun et al. 2015). A total of 33 QTLs for four seed dormancy parameters were identified in CSSLs, which were distributed on chromosomes 1, 2, 3, 4, 5, 8 and 11 (Fig. 2a). Among these QTLs, over 87% of the loci exhibited NIP alleles increasing seed dormancy (Additional file 7: Table S5). Seven to ten QTLs explained percentages of phenotypic variances ranging from 51.8% to 67.9%, respectively, for the germination parameters (G_3d_, AUC, T_50_ and G_7d_). For G_3d_, ten QTLs were detected, among which *qG*_*3d*_*3.3* on chromosome 3 had the most significant effect, explaining 18.9% of the phenotypic variance. For AUC, nine QTLs were identified, among which *qAUC3.3* and *qAUC11.2* had the most dominant effects, explaining 14.2% and 8.3% of the phenotypic variance, respectively. For T_50_, the major locus *qT*_*50*_*3.3* explained 17.0% of the phenotypic variance. *qG*_*7d*_*3.1* and *qG*_*7d*_*3.3* exhibited the most significant effect on G_7d_ out of seven QTLs, explaining 11.8% and 24.1% of the phenotypic variance, respectively. Among the identified QTLs, seven were found to affect three or more parameters simultaneously (Additional file 8: Table S6). These hotspot or clustered QTLs for multiple germination parameters were definitively designated as QTLs for seed dormancy. A total of four QTL overlapping regions (*qSD1.3*, *qSD5.1*, *qSD5.2*, *qSD11.1*) were shared among three parameters. Furthermore, the *qSD3.1* region located in Bin97 (0.39–0.70 Mb) and *qSD3.2* in Bin124 (27.40–28.49 Mb) of chromosome 3, *qSD8.2* in Bin278 (22.85–25.08 Mb) of chromosome 8 and *qSD11.2* in Bin361 (23.24–23.86 Mb) of chromosome 11 were associated with all four parameters, which is in accordance with the high correlations among these parameters (Additional file 4: Fig. S2). These data indicate that 12 QTL regions are associated with seed dormancy, among which at least four exhibit a major effect on the germination rate and germination uniformity in CSSLs.

### Detection of QTLs for Seed Dormancy in BILs

The QTLs detected in the BILs are summarized in Table S5. A total of 74 QTLs were identified for four seed germination parameters, which were distributed on all chromosomes except chromosome 12 (Fig. 2b). Most of these QTLs (78%) exhibited NIP alleles increasing seed dormancy. Fourteen to twenty-two QTLs explained 45.4% to 62.4% of the total phenotypic variance in the four parameters (Additional file 7: Table S5). For G_3d_, *qG*_*3d*_*3.3* had the most significant effect, explaining 10.2% of the phenotypic variance. For AUC, *qAUC3.3* presented the most significant effect, explaining 9.5% of the phenotypic variance. For T_50_, the major locus *qT*_*50*_*3.3* explained 8.9% of the phenotypic variance. For G_7d_, *qG*_*7d*_*6.1* was the major locus, accounting for 14.1% of the phenotypic variance. A total of 27 QTLs were commonly identified for various germination parameters, and eight overlapping QTL regions (*qSD2*, *qSD5.1*, *qSD5.2*, *qSD6.1*, *qSD8.1*, *qSD10*, *qSD11.2*, *qSD11.3*) shared among three parameters. Eight bins containing the QTL regions were detected for all four assayed parameters: for example, *qSD3.1* resided in Bin794 (0.35–0.59 Mb) and *qSD3.2* in Bin1033 (27.99–28.26 Mb) of chromosome 3. *qSD6.2* was located in Bin1752 (9.30–9.44 Mb) and *qSD6.3* in Bin1800 (20.80–20.96 Mb) of chromosome 6 (Additional file 7: Table S5). These overlapping QTL are consistent with the high correlations among the tested parameters in the BILs.

A comparison of the QTLs detected in the CSSLs and BILs revealed that a total of eight QTLs colocalized to the same or overlapping regions in both populations (Additional file 7: Table S5). The NIP alleles of these colocalized QTLs all increased seed dormancy in both populations. Among them, four QTL regions were identified for three or more parameters in both CSSLs and BILs (Additional file 8: Table S6). The first was the *qSD3.2* region, which showed major effects on the tested parameters and explained 4.6% to 24.1% of the phenotypic variance, with an average of 13.4%. The second was *qSD3.1* for seed dormancy, which explained 11.9% of the phenotypic variance in G_7d_. The third region, *qSD5.2*, was located on chromosome 5, and the fourth cluster, *qSD11.2*, was located on chromosome 11, both of which showed a moderate effect on seed dormancy (Additional file 7: Table S5). These colocalized QTLs associated with all assayed germination parameters in both populations indicate that QTL detection results were robust. Thus, these major loci were further investigated to dissect the genetic effects on seed dormancy.

### Validation of *qSD3.2* and *qSD3.1*

**Fig. 3:**
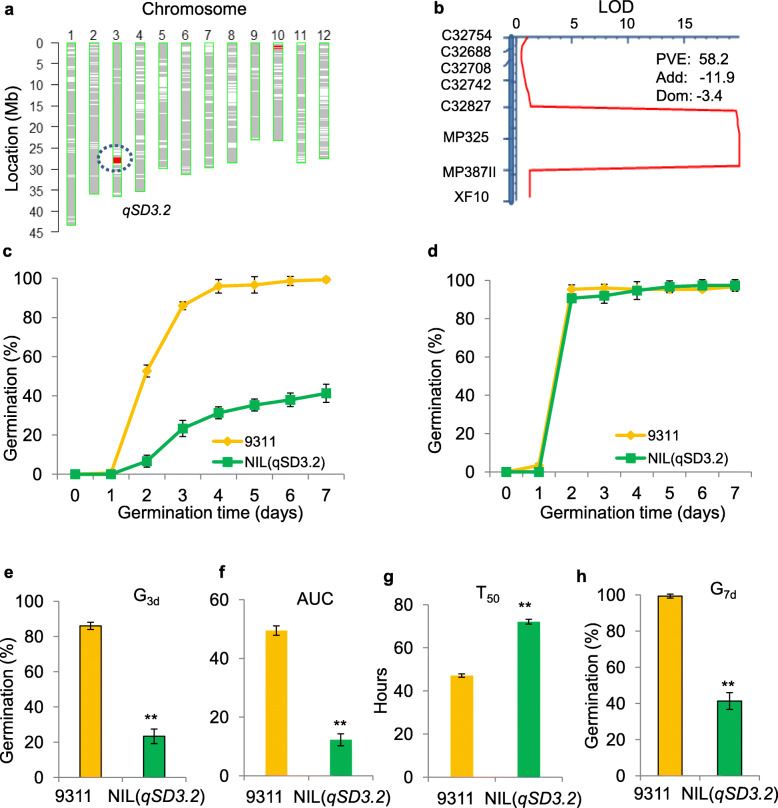
Validation of the effect of *qSD3.2* on seed dormancy. **a** Graphical genotype showing one CSSL (NY38) carrying an introduced Nipponbare segment encompassing *qSD3.2* on chromosome 3 and a background Nipponbare segment on chromosome 10 in the 9311 background. **b** QTLs detected in the NY38-derived population (*n* = 105). LOD, logarithm of odds; Add, additive effect; Dom, dominance effect; PVE, phenotypic variance explained by the QTL. **c** Germination curve of freshly harvested seeds of NIL (*qSD3.2*) and 9311. **d** Germination curve of NIL (*qSD3.2*) and 9311 seeds after dormancy breaks. **e-h** Germination behaviors of NIL (*qSD3.2*) and 9311 seeds. G_3d_ (**e**), AUC (**f**), T_50_ (**g**) and G_7d_ (**h**). G_3d_, germination rate at 72 h after imbibition; G_7d_, maximum germination rate at 168 h after imbibition; T_50_, germination speed, which is the time to reach 50% germination of seeds; and AUC, the area under the curve until 168 h after imbibition. The error bar represents the mean ± SD (*n* = 3). Double asterisks denote significant differences at *P* < 0.01

To validate the seed dormancy effect of *qSD3.2*, one CSSL (NY38) that carried an introduced NIP segment of *qSD3.2* on chromosome 3 and a background NIP segment on chromosome 10 was selected and crossed with 9311 to produce the NY38-derived population (Fig. 3a). The F_2_ population (*n* = 105) was genotyped using eight polymorphic markers (Additional file 2: Table S1) that covered the *qSD3.2* region and one polymorphic marker (ID1001) in the introduced segment of chromosome 10. QTL analysis in the F_2_ population confirmed that *qSD3.2* was located in the interval between C32827 and MP387II (approximately 110 kb), explaining 58.2% of the phenotypic variance for G_3d_. The additive effect of the locus was − 11.9, and the dominant effect was − 3.4 (Fig. 3b), which was in agreement with the much lower germination rate in the homozygous or heterozygous NIP genotype than in the 9311 genotype. In addition, single-point analysis using the ID1001 marker revealed that the region on chromosome 10 was not significantly associated with G_3d_ in the NY38-derived population (*P* = 0.4). These results indicated that *qSD3.2* is the major QTL underlying seed dormancy. Then, a near-isogenic line that harbored a single introduced NIP segment (from 26.80 Mb to 28.40 Mb) containing *qSD3.2* was developed from the population and designated as NIL (*qSD3.2*). NIL (*qSD3.2*) showed significantly lower G_3d_, AUC and G_7d_ values but a higher T_50_ than 9311 (Fig. 3e-h), suggesting that NIL (*qSD3.2*) could significantly delay seed germination and decrease germination uniformity compared with 9311. Furthermore, the after-ripened seeds from NIL (*qSD3.2*) and 9311 exhibited similar high germination rates when seed dormancy was broken (Fig. 3d), indicating that *qSD3.2* is a seed dormancy locus at which the NIP alleles increase seed dormancy.

**Fig. 4:**
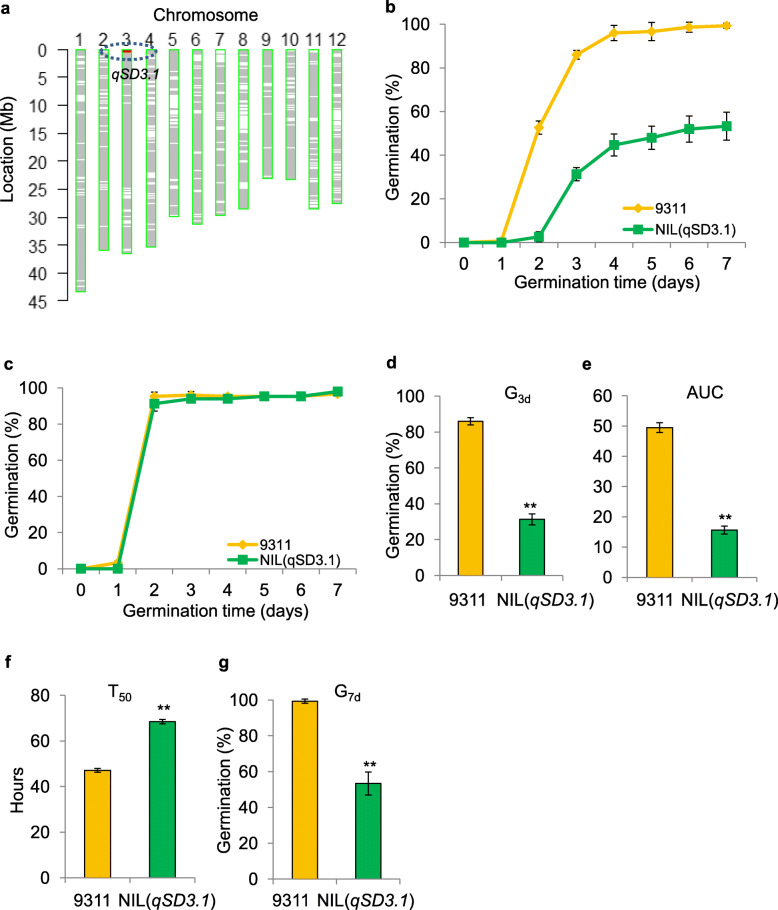
Validation of the effect of *qSD3.1* on seed dormancy. **a** Graphical genotype showing NIL (*qSD3.1*) that carries a single introduced Nipponbare segment encompassing *qSD3.1*. The red and green bars represent the Nipponbare segment and the 9311 background, respectively. **b** Germination curve of freshly harvested seeds of NIL (*qSD3.1*) and 9311. **c** Germination curve of NIL (*qSD3.1*) and 9311 seeds after dormancy breaks. **d-g** Germination behaviors of NIL (*qSD3.1*) and 9311; G_3d_ (**d**), AUC (**e**), T_50_ (**f**) and G_7d_ (**g**). G_3d_, germination rate at 72 h after imbibition; G_7d_, maximum germination rate at 168 h after imbibition; T_50_, germination speed, which is the time to reach 50% germination of seeds; and AUC, the area under the curve up to 168 h after imbibition. The error bar represents the mean ± SD (*n* = 3). Double asterisks indicate significant differences at *P* < 0.01

To validate the effect of *qSD3.1* on seed dormancy, NIL (*qSD3.1*), carrying a single introduced NIP segment (approximately 600 kb), was also developed from the F_2_ population (Fig. 4a). NIL (*qSD3.1*) showed significantly delayed germination compared with 9311 (Fig. 4b). After treatment at 43 °C for 3 days to relieve seed dormancy, the after-ripened seeds of NIL (*qSD3.1*) showed a similar germination rate to those of 9311 (Fig. 4c), confirming that *qSD3.1* truly affects seed dormancy. Accordingly, NIL (*qSD3.1*) seeds showed significantly lower values of the parameters of G_3d_, AUC and G_7d_ but a significantly higher T_50_ than 9311 seeds (Fig. 4d-g). These results indicate that the NIP alleles of *qSD3.1* contribute to high seed dormancy.

**Fig. 5:**
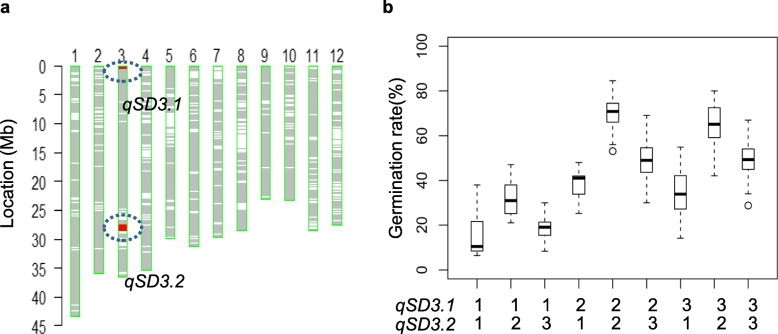
Epistatic interaction of *qSD3.1* and *qSD3.2*. **a** Graphical genotype of one CSSL (NY61) encompassing *qSD3.1* and *qSD3.2*. The red and green bars represent the Nipponbare (NIP) and 9311 genotypes, respectively. **b** Boxplots of nine genotypes at the two loci for germination rate at 72 h after imbibition in the NY61-derived population (*n* = 175). Box edges indicate the range of the 25th to 75th percentiles, with the median value shown by the bold middle line. “1”, “2” and “3” represent the NIP, 9311 and heterozygous genotypes, respectively

To determine the interaction between *qSD3.1* and *qSD3.2*, one CSSL line (NY61) carrying two introduced NIP segments containing both *qSD3.1* and *qSD3.2* was selected and crossed with 9311 to generate an NY61-derived population. In this F_2_ population (*n* = 175), two markers, MP30026 and C32827, which were tightly linked with *qSD3.1* and *qSD3.2,* respectively were used to classify nine genotypes at the two loci (Fig. 5a). Two-way analysis of the G_3d_ variance in the nine genotypes revealed that the two loci individually have significant effects on seed dormancy (Additional file 9: Table S7)*.* These results are consistent with QTL validation in the NILs as described above. Furthermore, *qSD3.1* and *qSD3.2* showed a significant digenic interaction (*P* < 0.02) (Additional file 9: Table S7). The genotypes that carried the NIP alleles at both loci exhibited the lowest G_3d_ (19.1%), and those carrying the 9311 alleles showed the highest G_3d_ (72.1%) (Fig. 5b). These results indicate that *qSD3.1* and *qSD3.2* may affect seed dormancy in rice through a synergistic interaction.

### ABA Sensitivity of NIL (*qSD3.1*) and NIL (*qSD3.2*)

It has been reported that ABA plays an essential role in the regulation of seed dormancy (Finkelstein et al. 2008; Liu et al. 2011). To investigate the ABA sensitivity of NIL (*qSD3.1*) and NIL (*qSD3.2*), after-ripened seeds of the NILs and 9311 were germinated under a series of exogenous ABA treatments (Additional file 10: Fig. S3). Compared with the 9311 seeds, the NIL (*qSD3.1*) and NIL (*qSD3.2*) seeds showed no significant effect on G_7d_ under a low concentration of ABA (up to 10 μM), whereas a significant decrease in G_7d_ was observed under both 20 and 30 μM ABA. The ABA sensitivity of the NILs suggests that both *qSD3.1* and *qSD3.2* may be associated with ABA-responsive genes.

## Discussion

Seed dormancy is a complex quantitative trait controlled by multiple genes. The identification and characterization of the QTLs for seed dormancy is essential for understanding the genetic basis of seed dormancy in crops. In the present study, we identified a large number of QTLs for seed dormancy in CSSL and BIL populations that were derived from common parental lines (NIP and 9311) and genotyped by using high-density SNP markers (Fig. 2). A comparison of the QTLs detected in the CSSLs and BILs revealed many more QTLs (27 versus 12) in the BILs than in the CSSLs because more SNP markers were developed via GBS strategy for the relatively large BIL population. In addition, 19 QTLs were only detected in BILs and four loci were identified only in CSSLs (Additional file 7: Table S5). Specifically, *qG*_*3d*_*1.3*, *qAUC8.2*, *qT*_*50*_*2.2* and *qG*_*7d*_*4.2* were detected in CSSLs, but not in BILs, suggesting that they may be specific and may not be affected by the interaction effect of other loci in a similar genetic background. In contrast, *qG*_*3d*_*4.1*, *qAUC4*, *qT*_*50*_*4* and *qG*_*7d*_*4* were only identified in BILs, indicating the possible occurrence of epistatic interaction in BILs due to more background introgressions. Therefore, the integration of the CSSLs and BILs with a high-density bin map is an effective strategy for elucidating the genetic architecture of a complex trait such as seed dormancy.

It is notable that eight QTLs were commonly identified in the two populations. Among these QTLs, four loci (*qSD3.1*, *qSD3.2*, *qSD5.2* and *qSD11.2*) were found to have major effects on seed dormancy, with the NIP alleles increasing seed dormancy (Additional file 7: Table S5). The effects of the two loci (*qSD3.1* and *qSD3.2*) and their epistatic interaction on seed dormancy were further validated and identified by using a CSSL-derived segregating population (Figs. 3, 4, 5; Additional file 9: Table S7). These data indicate that the major QTLs, along with epistatic interactions, play a crucial role in seed dormancy, as reported in other studies (Wang et al. 2013). Considering the ABA sensitivity of the NILs carrying the corresponding *qSD3.1* and *qSD3.2* loci (Additional file 10: Fig. S3), we propose that the epistatic interaction of *qSD3.1* and *qSD3.2* may affect seed dormancy through the ABA pathway (Graeber et al. 2012).

In the present study, a high-density linkage map was developed in a relatively large NIP × 9311 BIL population; this map harbors 3235 bins with an average physical interval of 115 kb. Selected bins can be used to identify QTLs in a smaller bin region from which potential candidate genes in the peak bin may be suggested using the gene annotation database (http://rice.plantbiology.msu.edu/). In this case, nine putative candidate genes were found in the peak bin (approximately 238 kb) for *qSD3.1*. Five out of the nine genes were specifically and highly expressed in the seeds (Additional file 11: Table S8) and were significantly induced or repressed by ABA treatment (http://tenor.dna.affrc.go.jp). These include RNA methyltransferase (LOC_Os03g01110), the known gene *qLTG3–1* (LOC_Os03g01320), associated with low-temperature germinability (Fujino et al. 2008), and genes encoding phosphatase-2c (LOC_Os03g01365), a tubulin domain-containing protein (LOC_Os03g01530), and a DNA-binding protein (LOC_Os03g01540). Recently, it has been reported that the DNA-binding protein AT-Hook-Like 10 is involved in the ABA signaling pathway for drought stress in *Arabidopsis* (Wong et al. 2019). Intriguingly, the seed dormancy gene *DOG1* requires the phosphatases from the ABA signaling pathway to control seed dormancy in *Arabidopsis* (Née et al. 2017a, 2017b). The data suggest that these five genes are the most likely candidate genes for *qSD3.1*. However, other genes surrounding the peak bin of *qSD3.1* that have been reported to be associated with seed dormancy or seed storability should not be excluded from consideration, such as the seed dormancy gene *OsG* (Wang et al. 2018) and the fatty acid hydroxylase gene *OsFAH2* affecting seed storability (Yuan et al. 2019). For *qSD3.2*, eight out of seventeen putative genes in its peak bin (approximately 268 kb) can be considered putative candidates, as they were specifically and highly expressed in the seeds and significantly induced or repressed by ABA. Among these genes, three genes located in the peak bin of *qSD3.2*, including the preharvest sprouting locus *qPHS3* reported in a previous study (Suzuki et al. 2015), a lipoxygenase gene (*OsLOX3*) associated with seed longevity (Xu et al. 2015) and an ethylene-related gene (LOC_Os03g49400) are of interest. It has been reported that *ethylene insensitive2*, a homologous gene of LOC_Os03g49400, can increase ABA sensitivity during seed germination in *Arabidopsis* (Wang et al. 2007). Thus, the two major QTLs identified in the present study could be set as a priority for the identification of the causal genes and the characterization of their functional relevance to seed dormancy.

In addition, by comparing the QTLs detected in the current study with those identified in other studies, we found that at least 13 QTLs colocalized in the same or overlapping regions that harbor genes and/or loci known to be associated with seed dormancy (Fang et al. 2008; Sugimoto et al. 2010; Ye et al. 2015; Wang et al. 2018). For example, *qT*_*50*_*1.4* detected in BILs is located near *sd1*, which has been reported to regulate seed dormancy (Ye et al. 2015). *qAUC7/qT*_*50*_*7* is localized near the seed dormancy gene *Sdr4* (Sugimoto et al. 2010). *qG*_*7d*_*3.2* is mapped near the phytoene desaturase gene *OsPDS* for seed dormancy (Fang et al. 2008). *qSD1.3* and *qSD5.1* were detected in QTL regions related to seed dormancy in a previous report (Magwa et al. 2016). *qSD6.2*, *qSD6.3* and *qSD8.2* were mapped in previously reported QTL regions for seed dormancy (Marzougui et al. 2012). We also identified 26 novel loci that have not been reported previously. In this regard, *qSD5.2* for seed dormancy was found in an approximately 30-kb peak bin region on chromosome 5, and *qSD11.2* was located in an approximately 33-kb peak bin region on chromosome 11 in both populations (Additional file 6: Table S4). The peak bin of *qSD5.2* contained two genes (LOC_Os05g50110 and LOC_Os05g50120). As LOC_Os05g50110 was significantly upregulated by ABA treatment, it represents a promising candidate gene. *qSD11.2*, includes only one gene (LOC_Os11g39020), encoding an ABC transporter/ATP-binding protein that is significantly induced by ABA. The ABC transporter *peroxisomal membrane protein 2* has been reported to be associated with seed germination in *Arabidopsis* (Verrier et al. 2008). Thus, the novel QTLs with a fine resolution encompassing a small number of candidate genes could be further functionally analyzed and immediately exploited for the improvement of appropriate dormancy through marker-assisted selection. Importantly, SNP genotyping revealed that the CSSLs have only one or a few introduced NIP segments in the same background of 9311, which provides an efficient strategy for developing secondary populations segregating at the QTL of interest for validation, fine mapping and functional analyses. Therefore, the developed CSSLs and BILs together with the major QTLs detected in the present study can be utilized for the further map-based cloning of the causal genes for seed dormancy.

## Conclusions

The present study identified 31 QTLs for seed dormancy using CSSL and BIL populations with high-density SNP maps that were developed from common parents. Among these QTLs, 13 QTL regions contain genes that were previously associated with seed germination or seed dormancy. Eight major QTLs for seed dormancy were commonly identified in both the populations. Moreover, two major loci (*qSD3.1* and *qSD3.2*) and their digenic interaction for seed dormancy were further validated in two CSSL-derived secondary populations. The NILs that carry either *qSD3.1* or *qSD3.2* are sensitive to ABA, suggesting that *qSD3.1* and *qSD3.2* may affect seed dormancy through the ABA pathway. These findings would be helpful for identifying candidate genes and characterizing the molecular mechanisms underlying seed dormancy. The current data demonstrate that the BILs integrated with the CSSLs derived from the common parents are an excellent resource facilitating the identification and characterization of loci for complex traits such as seed germination and dormancy at a fine scale. The identification of the major QTLs with putative candidate genes associated with seed germination behaviors in this study lays a foundation for cloning the causal genes of this complex trait and will facilitate the improvement of appropriate seed dormancy by genomic selection approaches.

## Supplementary information

**Additional file 1: Figure S1.** Flowchart of the development of CSSLs and BILs derived from the common parents Nipponbare (NIP) and 9311. CSSLs, chromosome segment substitution lines; BILs, backcross inbred lines.

**Additional file 2: Table S1.** Primers used in this study to genotype the NY38- and NY61-derived F_2_ populations.

**Additional file 3: Table S2.** Seed dormancy of the parents Nipponbare and 9311, CSSLs and BILs.

**Additional file 4: Figure S2.** Correlation coefficients among G_3d_, AUC, T_50_ and G_7d_ in CSSLs (**a**) and BILs (**b**). G_3d_, germination rate at 72 h after imbibition; G_7d_, maximum germination rate at 168 h after imbibition; T_50_, germination speed, which is the time to reach 50% germination of seeds; and AUC, the area under the curve up to 168 h after imbibition. The upper panel contains the correlation coefficients and the lower panel contains the frequency distribution of the assayed parameters. The diagonal represents the histogram of the traits. Double asterisks represent significance at *P* < 0.01.

**Additional file 5: Table S3.** Bin map information of Nipponbare × 9311 CSSLs.

**Additional file 6: Table S4.** Bin map information of Nipponbare × 9311 BILs.

**Additional file 7: Table S5.** QTLs detected for four seed dormancy parameters in CSSLs and BILs derived from the common parents of Nipponbare and 9311.

**Additional file 8: Table S6.** QTLs detected in the same or overlapping regions for seed dormancy in CSSLs and BILs.

**Additional file 9: Table S7.** Epistatic interaction of *qSD3.1* and *qSD3.2* in the NY61-derived population.

**Additional file 10: Fig. S3.** ABA sensitivity of the after-ripened seeds of NIL (*qSD3.1*), NIL (*qSD3.2*) and 9311. The germination rate on the *y*-axis indicates the maximum germination rate at 168 h after imbibition. Error bar represents the mean ± SD (*n* = 3).

**Additional file 11: Table S8.** The putative candidate genes for four major loci: *qSD3.1*, *qSD3.2*, *qSD5.2* and *qSD11.2*.

## Data Availability

The data sets supporting the results of this article are included within the article and its supporting files.

## Abbreviations

AUC: The area under the curve up to 168 h after imbibition
BILs: Backcross inbred lines
CSSLs: Chromosome segment substitution lines
GBS: Genotyping by sequencing
LOD: Logarithm of odds
NIL: Near-isogenic line
PVE: Phenotypic variance explained
QTLs: Quantitative trait loci
SNP: Single nucleotide polymorphism

## References

[CR1] Anderson JA, Sorrells ME, Tanksley SD (1993). RFLP analysis of genomic regions associated with resistance to preharvest sprouting in wheat. Crop Sci.

[CR2] Bentsink L, Jowett J, Hanhart CJ, Koornneef M (2006). Cloning of *DOG1*, a quantitative trait locus controlling seed dormancy in Arabidopsis. Proc Natl Acad Sci U S A.

[CR3] Cai HW, Morishima H (2000). Genomic regions affecting seed shattering and seed dormancy in rice. Theor Appl Genet.

[CR4] Cantoro R, Fernández LG, Cervigni GDL, Rodríguez MV, Gieco JO, Paniego N, Heinz RA, Benech-Arnold RL (2016). Seed dormancy QTL identification across a sorghum bicolor segregating population. Euphytica.

[CR5] Chen M, Penfield S (2018). Feedback regulation of *COOLAIR* expression controls seed dormancy and flowering time. Science.

[CR6] Fang J, Chai CL, Qian Q, Li CL, Tang JY, Sun L, Huang ZJ, Guo XL, Sun CH, Liu M, Zhang Y, Lu QT, Wang YQ, Lu CM, Han B, Chen F, Cheng ZK, Chu CC (2008). Mutations of genes in synthesis of the carotenoid precursors of ABA lead to pre-harvest sprouting and photo-oxidation in rice. Plant J.

[CR7] Finkelstein R, Reeves W, Ariizumi T, Steber C (2008). Molecular aspects of seed dormancy. Annu Rev Plant Biol.

[CR8] Fujino K, Sekiguchi H, Matsuda Y, Sugimoto K, Ono K, Yano M (2008). Molecular identification of a major quantitative trait locus, *qLTG3-1*, controlling low-temperature germinability in rice. Proc Natl Acad Sci U S A.

[CR9] Graeber K, Linkies A, Steinbrecher T, Mummenhoff K, Tarkowska D, Tureckova V, Ignatz M, Sperber K, Voegele A, Jong H, Urbanová T, Strnad M, Leubner-Metzger G (2014). *DELAY OF GERMINATION 1* mediates a conserved coat-dormancy mechanism for the temperature- and gibberellin-dependent control of seed germination. Proc Natl Acad Sci U S A.

[CR10] Graeber K, Nakabayashi K, Miatton E, Leubner-Metzger G, Soppe W (2012). Molecular mechanisms of seed dormancy. Plant Cell Environ.

[CR11] Gu XY, Foley ME, Horvath DP, Anderson JV, Feng JH, Zhang LH, Mowry CR, Ye H, Suttle JV, Kadowaki KI, Chen ZX (2011). Association between seed dormancy and pericarp color is controlled by a pleiotropic gene that regulates abscisic acid and flavonoid synthesis in weedy red rice. Genetics.

[CR12] Gu XY, Liu TL, Feng JH, Jeffrey CS, Gibbons J (2010). The *qSD12* underlying gene promotes abscisic acid accumulation in early developing seeds to induce primary dormancy in rice. Plant Mol Biol.

[CR13] Huang XH, Feng Q, Qian Q, Zhao Q, Wang L, Wang A, Guan JP, Fan DL, Weng QJ, Huang T, Dong J, Sang T, Han B (2009). High-throughput genotyping by whole-genome resequencing. Genome Res.

[CR14] Joosen RV, Kodde J, Willems LJ, Ligterink W, Linus HW, Henk WM (2010). GERMINATOR: a software package for high-throughput scoring and curve fitting of *Arabidopsis* seed germination. Plant J.

[CR15] Li GW, Li XT, Wang Y, Mi JM, Xing F, Zhang DH, Dong YY, Li XH, Xiao JH, Zhang QF, Ouyang YD (2017). Three representative inter and intra-subspecific crosses reveal the genetic architecture of reproductive isolation in rice. Plant J.

[CR16] Li M, Sun PL, Zhou HJ, Chen S, Yu SB (2011). Identification of quantitative trait loci associated with germination using chromosome segment substitution lines of rice (*Oryza sativa* L.). Theor Appl Genet.

[CR17] Lin SY, Sasaki T, Yano M (1998). Mapping quantitative trait loci controlling seed dormancy and heading date in rice, *Oryza sativa* L. using backcross inbred lines. Theor Appl Genet.

[CR18] Liu F, Zhang HX, Wu G, Sun J, Hao LL, Ge XM, Yu J, Wang WW (2011). Sequence variation and expression analysis of seed dormancy- and germination-associated ABA- and GA-related genes in rice cultivars. Front Plant Sci.

[CR19] Lu Q, Niu XJ, Zhang MC, Wang CH, Xu Q, Feng Y, Yang YL, Wang S, Yuan XP, Yu HY, Wang YP, Chen XP, Liang XQ, Wei XH (2018). Genome-wide association study of seed dormancy and the genomic consequences of improvement footprints in rice (Oryza sativa L.). Front Plant Sci.

[CR20] Magwa RA, Zhao H, Xing YZ (2016). Genome-wide association mapping revealed a diverse genetic basis of seed dormancy across subpopulations in rice (Oryza sativa L.). BMC Genet.

[CR21] Marzougui S, Sugimoto K, Yamanouchi U, Shimono M, Hoshino T, Hori K, Kobayashi M, Ishiyama K, Yano M (2012). Mapping and characterization of seed dormancy QTLs using chromosome segment substitution lines in rice. Theor Appl Genet.

[CR22] McCouch SR (2008). Gene nomenclature system for rice. Rice.

[CR23] Meng L, Li HH, Zhang LY, Wang JK (2015). QTL IciMapping: integrated software for genetic linkage map construction and quantitative trait locus mapping in biparental populations. Crop J.

[CR24] Mizuno Y, Yamanouchi U, Hoshino T, Nonoue Y, Nagata K, Fukuoka S, Ando T, Yano M, Sugimoto K (2018). Genetic dissection of pre-harvest sprouting resistance in an upland rice cultivar. Breed Sci.

[CR25] Müller K, Bouyer D, Schnittger A, Kermode AR (2012). Evolutionarily conserved histone methylation dynamics during seed life-cycle transitions. PLoS One.

[CR26] Nagel M, Alqudah AM, Bailly M, Rajjou L, Pistrick S, Matzig G, Börner A, Kranner I (2019). Novel loci and a role for nitric oxide for seed dormancy and preharvest sprouting in barley. Plant Cell Environ.

[CR27] Née G, Kramer K, Nakabayashi K, Yuan BJ, Xiang Y, Miatton E, Finkemeier I, Soppe W (2017). *DELAY OF GERMINATION1* requires PP2C phosphatases of the ABA signalling pathway to control seed dormancy. Nat Commun.

[CR28] Née G, Xiang Y, Soppe WJ (2017). The release of dormancy, a wake-up call for seeds to germinate. Curr Opin Plant Biol.

[CR29] Nguyen T, Zhou CL, Zhang TY, Yu JF, Miao R, Huang YS, Zhu XJ, Song WH, Liu X, Mou CL, Lan L, Liu SJ, Tian YL, Zhao ZG, Jiang L, Wan JM (2019). Identification of QTL for seed dormancy from weedy rice and its application to elite rice cultivar ‘Ninggeng 4’. Mol Breed.

[CR30] Schatzki J, Schoo B, Ecke W, Herrfurth C, Feussner I, Becker HC, Möllers C (2013). Mapping of QTL for seed dormancy in a winter oilseed rape doubled haploid population. Theor Appl Genet.

[CR31] Shu K, Liu XD, Xie Q, He ZH (2016). Two faces of one seed: hormonal regulation of dormancy and germination. Mol Plant.

[CR32] Sugimoto K, Takeuchi Y, Ebana K, Miyao A, Hirochika H, Hara H, Ishiyama K, Kobayashi M, Ban Y, Hattori T, Yano M (2010). Molecular cloning of *Sdr4*, a regulator involved in seed dormancy and domestication of rice. Proc Natl Acad Sci U S A.

[CR33] Sun WQ, Zhou Q, Yao Y, Qiu XJ, Xie K, Yu SB (2015). Identification of genomic regions and the isoamylase gene for reduced grain chalkiness in rice. PLoS One.

[CR34] Suzuki Y, Miura K, Shigemune A, Sasahara H, Ohta H, Uehara Y, Ishikawa T, Hamada S, Shirasawa K (2015). Marker-assisted breeding of a LOX-3-null rice line with improved storability and resistance to preharvest sprouting. Theor Appl Genet.

[CR35] Tan CJ, Sun YJ, Xu HS, Yu SB (2011). Identification of quantitative trait locus and epistatic interaction for degenerated spikelets on the top of panicle in rice. Plant Breed.

[CR36] Verrier PJ, Bird D, Burla B, Dassa E, Forestier C, Geisler M, Klein M, Kolukisaoglu U, Lee Y, Martinoia E, Murphy A, Rea PA, Samuels L, Schulz B, Spalding EJ, Yazaki K, Theodoulou FL (2008). Plant ABC proteins–a unified nomenclature and updated inventory. Trends Plant Sci.

[CR37] Wan JM, Jiang L, Tang JY, Wang CM, Hou MY, Jing W, Zhang LX (2006). Genetic dissection of the seed dormancy trait in cultivated rice (*Oryza sativa* L.). Plant Sci.

[CR38] Wang L, Cheng JP, Lai YY, Du WL, Huang X, Wang ZF, Zhang HS (2013). Identification of QTLs with additive, epistatic and QTL × development interaction effects for seed dormancy in rice. Planta.

[CR39] Wang M, Li WZ, Fang C, Xu F, Liu YC, Wang Z, Yang R, Zhang M, Liu SL, Lu SJ, Lin T, Tang JY, Wang YQ, Wang HR, Lin H, Zhu BG, Chen MS, Kong FJ, Liu BH, Zeng DL, Jackson SA, Chu CC, Tian ZX (2018). Parallel selection on a dormancy gene during domestication of crops from multiple families. Nat Genet.

[CR40] Wang YN, Liu C, Li KX, Sun FF, Hu HZ, Li X, Zhao YK, Han CY, Zhang WS, Duan YF, Liu MY, Li X (2007). Arabidopsis EIN2 modulates stress response through abscisic acid response pathway. Plant Mol Biol.

[CR41] Wong MM, Bhaskara GB, Wen TN, Lin WD, Nguyen TT, Chong GL, Verslues PE (2019). Phosphoproteomics of *Arabidopsis* highly ABA-induced1 identifies AT-hook-Like10 phosphorylation required for stress growth regulation. Proc Natl Acad Sci U S A.

[CR42] Xu HB, Wei YD, Zhu YS, Lian L, Xie HG, Cai QH, Chen QS, Lin ZP, Wang ZH, Xie HA, Zhang JF (2015). Antisense suppression of *LOX3* gene expression in rice endosperm enhances seed longevity. Plant Biotechnol J.

[CR43] Ye H, Feng J, Zhang L, Zhang J, Mispan MS, Cao Z, Beighley DH, Yang J, Gu XY (2015). Map-based cloning of *Seed Dormancy1-2* identified a gibberellin synthesis gene regulating the development of endosperm-imposed dormancy in rice. Plant Physiol.

[CR44] Yu HH, Xie WB, Li J, Zhou FS, Zhang Q (2014). A whole-genome SNP array (RICE6K) for genomic breeding in RICE. Plant Biotechnol J.

[CR45] Yu HH, Xie WB, Wang J, Xing YZ, Xu CG, Li XH, Xiao JH, Zhang Q (2011). Gains in QTL detection using an ultra-high density SNP map based on population sequencing relative to traditional RFLP/SSR markers. PLoS One.

[CR46] Yuan ZY, Fan K, Xia LF, Ding XL, Tian L, Sun WQ, He HZ, Yu SB (2019). Genetic dissection of seed storability and validation of candidate gene associated with antioxidant capability in rice (Oryza sativa L.). Int J Mol Sci.

