# Supplementary figures and images for: Genetic Dissection of Seed Dormancy in Rice (Oryza sativa L.) by Using Two Mapping Populations Derived from Common Parents
Source: Rice (N Y). 2020 Aug 5;13:52. doi: 10.1186/s12284-020-00413-4 (PMC7406625; doi:10.1186/s12284-020-00413-4)

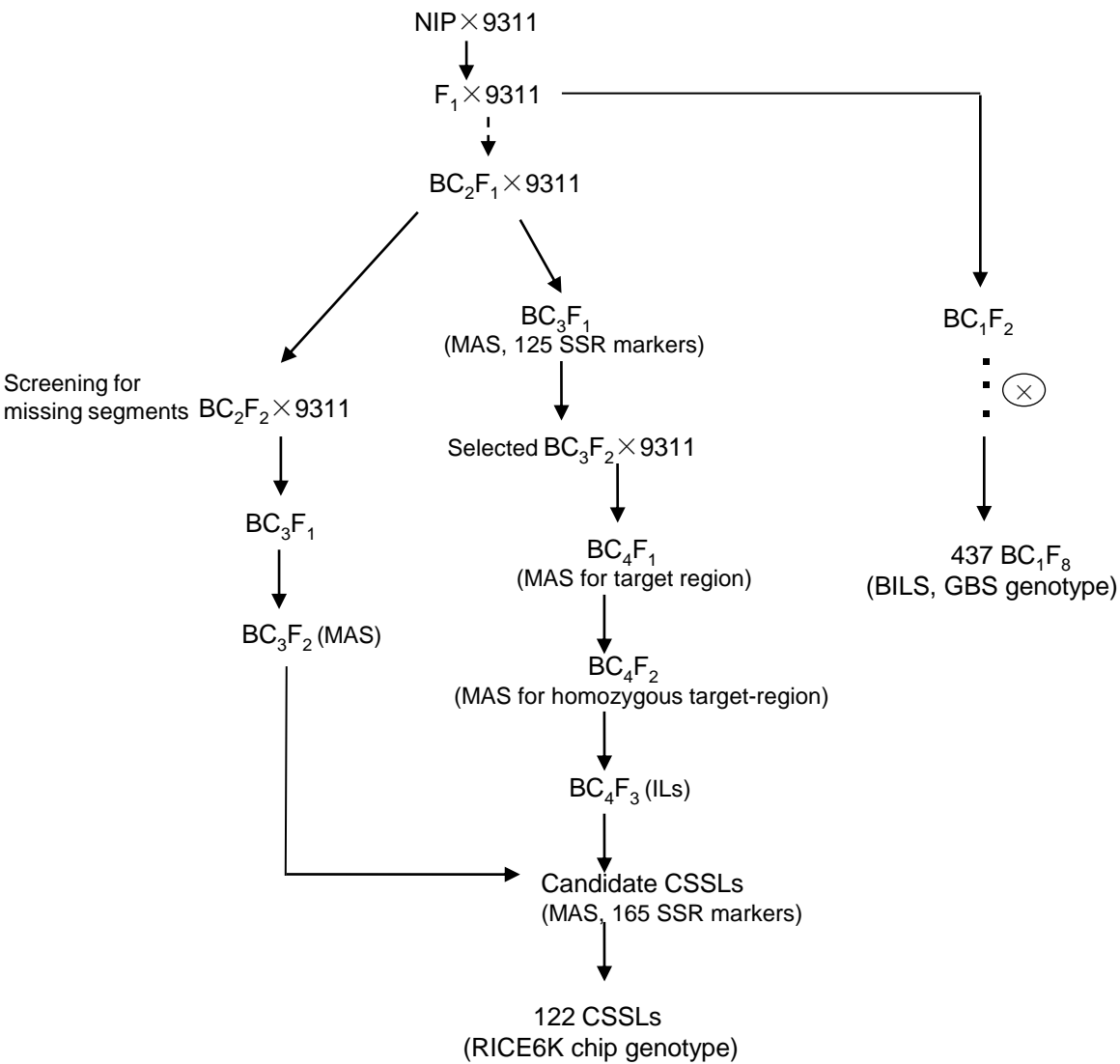

Supplement: Supplementary file 1 — Additional file 1: Figure S1. Flowchart of the development of CSSLs and BILs derived from the common parents Nipponbare (NIP) and 9311. CSSLs, chromosome segment substitution lines; BILs, backcross inbred lines. [file 12284_2020_413_MOESM1_ESM.pdf]

**a**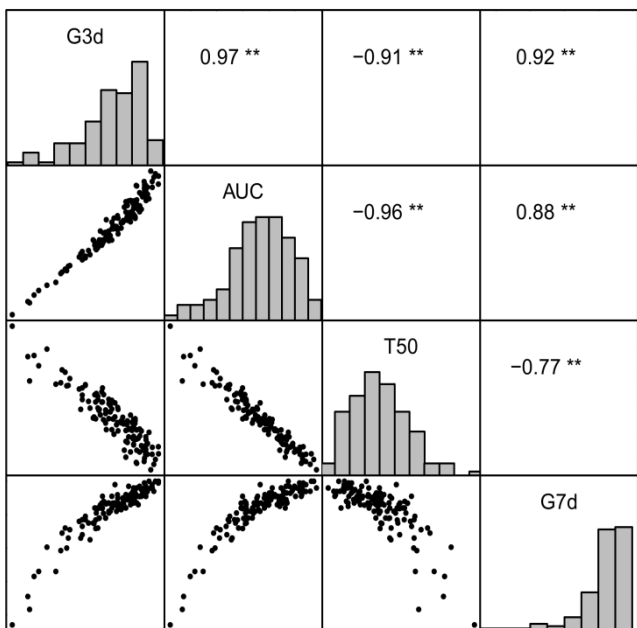**b**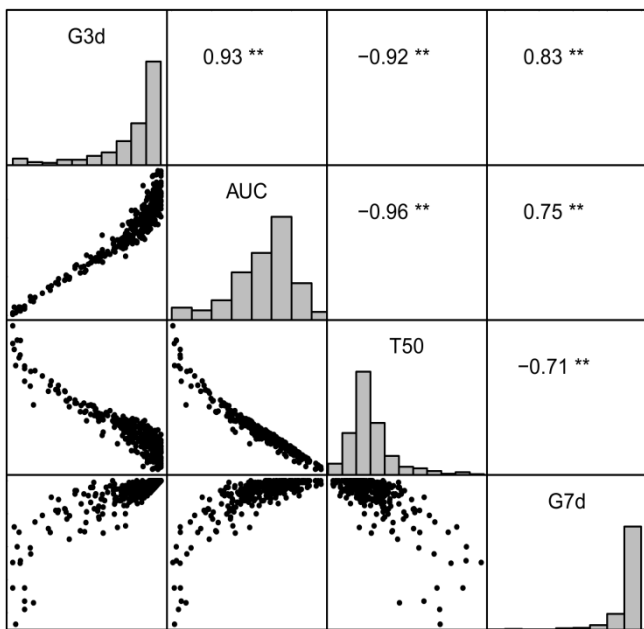

Supplement: Supplementary file 4 — Additional file 4: Figure S2. Correlation coefficients among G3d, AUC, T50 and G7d in CSSLs (a) and BILs (b). G3d, germination rate at 72 h after imbibition; G7d, maximum germination rate at 168 h after imbibition; T50, germination speed, which is the time to reach 50% germination of seeds; and AUC, the area under the curve up to 168 h after imbibition. The upper panel contains the correlation coefficients and the lower panel contains the frequency distribution of the assayed parameters. The diagonal represents the histogram of the traits. Double asterisks represent significance at P < 0.01. [file 12284_2020_413_MOESM4_ESM.pdf]

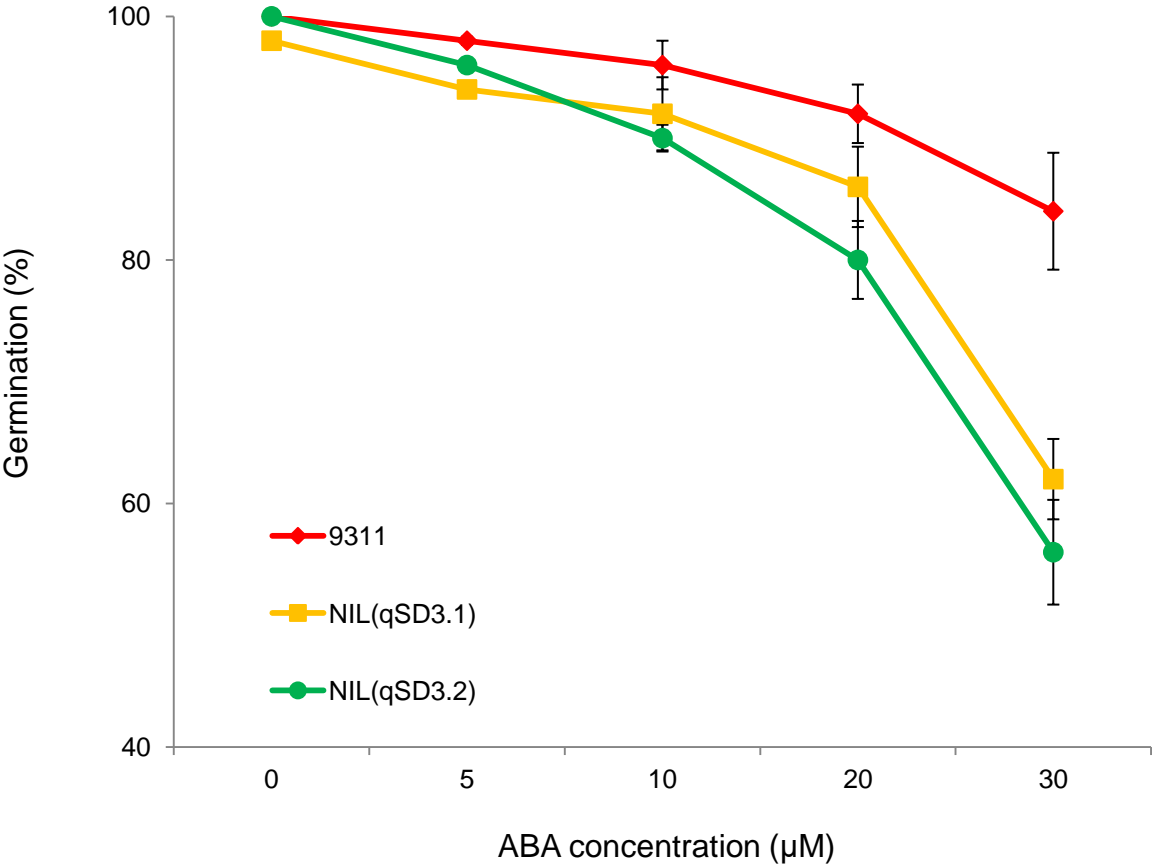

Supplement: Supplementary file 10 — Additional file 10: Fig. S3. ABA sensitivity of the after-ripened seeds of NIL (qSD3.1), NIL (qSD3.2) and 9311. The germination rate on the y-axis indicates the maximum germination rate at 168 h after imbibition. Error bar represents the mean ± SD (n = 3). [file 12284_2020_413_MOESM10_ESM.pdf]
