# Supplementary material for: Genetic Dissection of Seed Dormancy in Rice (Oryza sativa L.) by Using Two Mapping Populations Derived from Common Parents
Source: Rice (N Y). 2020 Aug 5;13:52. doi: 10.1186/s12284-020-00413-4 (PMC7406625; doi:10.1186/s12284-020-00413-4)
Supplement: Supplementary file 2 — Additional file 2: Table S1. Primers used in this study to genotype the NY38- and NY61-derived F2 populations. [file 12284_2020_413_MOESM2_ESM.docx]

**Table S1.** Primers used in this study to genotype the NY38- and NY61-derived F2 populations ^a^.

| Marker | Chr ^b^ | Position (Mb) ^c^ | Forward primer sequence (5'-3') | Reverse primer sequence (5'-3') | Purpose |
| --- | --- | --- | --- | --- | --- |
| C32688 | 3 | 26.88 | GTGTGCATAGAAAATTGAGC | TCAAAGCAAACTAAATCGGT | To map *qSD3.2* introgression |
| C32708 | 3 | 27.08 | GCTTATCCCAGATTCCAGTT | CAGGTATGGTTTTCCTTTTG |  |
| C32742 | 3 | 27.42 | TGGTGAAATTGGTAAAGTGTCA | GCAGTGGCTGATAGGTGGAC |  |
| C32754 | 3 | 27.54 | AAAACTCTGTACCTCCATCG | CAAGCATGAGATGAGTTCAA |  |
| XF10 | 3 | 28.12 | GCACACATAACAACTGCAAA | TCTACAGGCGAAGAAAATGA |  |
| MP387II | 3 | 28.15 | AGGGAGTGGCGACTAGTGTG | ACGTGGAGGCTCCTTTCTTT |  |
| C32827 | 3 | 28.27 | ACAATTCACAACTGGTGGTT | ACTACTACTCCTGGGCTCCT |  |
| MP325 | 3 | 28.28 | TTTAATGATAAATCAAGCCACA | GCGATGTAACCCTTTTCTTT |  |
| MP30026 | 3 | 0.33 | GCTGTTAAAATGAAGCGATAA | GACCACTAGGCACAAAATGT | To map *qSD3.1* introgression |
| ID1001 | 10 | 0.15 | ATGGGTATTGTCTCCCATGT | AGAAATCCTTCTAGCGATGC | To map chr 10 introgression |

^a^The NY38-derived population segregated for the *qSD3.1* introgression and the NY61-derived population for the *qSD3.2* introgression. ^b^ Chr, Chromosome. ^c^ Physical position of a given marker is based on the database MSU7.0 (http://rice.plantbiology.msu.edu/index.shtml).
