# Supplementary material for: Genetic Dissection of Seed Dormancy in Rice (Oryza sativa L.) by Using Two Mapping Populations Derived from Common Parents
Source: Rice (N Y). 2020 Aug 5;13:52. doi: 10.1186/s12284-020-00413-4 (PMC7406625; doi:10.1186/s12284-020-00413-4)
Supplement: Supplementary file 3 — Additional file 3: Table S2. Seed dormancy of the parents Nipponbare and 9311, CSSLs and BILs. [file 12284_2020_413_MOESM3_ESM.docx]

**Table S2.** Seed dormancy of Nipponbare and 9311, CSSLs and BILs

| Trait^a^ | Parents | | ^b^CSSLs | | ^c^BILs | |
| --- | --- | --- | --- | --- | --- | --- |
|  | Nipponbare | 9311 | Mean±SD^d^ | Range | Mean±SD | Range |
| G_3d_ | 12.6±3.1** | 86.0±2.0 | 68.3±19.5 | 3.0-97.0 | 80.2±23.8 | 0-100.0 |
| AUC | 7.2±1.9** | 49.5±1.6 | 36.9±11.6 | 2.2-58.2 | 46.3±15.2 | 0.5-74.8 |
| T_50_ | 91.4±3.7 ** | 47.1±0.8 | 54.9±9.2 | 37.7-86.7 | 50.2±16.1 | 23.5-128.0 |
| G_7d_ | 40.6±4.2** | 99.3±1.1 | 83.5±14.7 | 14.0-100.0 | 92.6±11.9 | 13.0-100.0 |

^a^ G_3d_, germination rate at 72 h after imbibition; G_7d_, maximum germination rate at 168 h after imbibition; T_50_, germination speed, which is the time to reach 50% germination of seeds; and AUC, area under the curve up to 168 h after imbibition. ^b^ CSSLs, chromosome segment substitution lines; ^c^ BILs, backcross inbred lines; ^d^ mean ± standard deviation. ** significance at *P*< 0.01.
