# Supplementary material for: Genetic Dissection of Seed Dormancy in Rice (Oryza sativa L.) by Using Two Mapping Populations Derived from Common Parents
Source: Rice (N Y). 2020 Aug 5;13:52. doi: 10.1186/s12284-020-00413-4 (PMC7406625; doi:10.1186/s12284-020-00413-4)
Supplement: Supplementary file 7 — Additional file 7: Table S5. QTLs detected for four seed dormancy parameters in CSSLs and BILs derived from the common parents of Nipponbare and 9311. [file 12284_2020_413_MOESM7_ESM.docx]

**Table S5.** QTLs detected for four seed dormancy parameters in CSSLs and BILs derived from the common parents of Nipponbare and 9311

| ^a^Trait | ^b^Chr | Interval (Mb) | ^c^QTL | CSSLs | | | BILs | | | Genes or QTLs reported |
| --- | --- | --- | --- | --- | --- | --- | --- | --- | --- | --- |
|  |  |  |  | *P* value | Effect | PVE (%) | *P* value | Effect | ^d^PVE (%) |  |
| G_3d_ | 1 | 17.95-17.99 | *qG_3d_1.1* |  |  |  | 2.3E-03 | 0.07 | 1.7 |  |
|  | 1 | 26.71-26.88 | *qG_3d_1.2* |  |  |  | 2.8E-04 | 0.13 | 0.2 |  |
|  | 1 | 30.4-33.3 | *qG_3d_1.3* | 1.1E-03 | 2.82 | 5.8 |  |  |  | *FHS1.1* |
|  | 2 | 7.38-7.41 | *qG_3d_2.1* |  |  |  | 8.9E-04 | -0.12 | 2.5 |  |
|  | 2 | 12.2-14.3 | *qG_3d_2.2* | 3.6E-03 | -4.3 | 3.3 |  |  |  |  |
|  | 3 | 0.05-0.70 | *qG_3d_3.1* | 5.9E-07 | -5.03 | 6.5 | 3.2E-10 | -0.27 | 7.2 | *OsG* |
|  | 3 | 14.38-14.5 | *qG_3d_3.2* |  |  |  | 1.6E-03 | -0.3 | 2.2 |  |
|  | 3 | 27.4-28.45 | *qG_3d_3.3* | 3.0E-10 | -6.03 | 18.9 | 3.2E-09 | -0.2 | 10.2 | *qPHS3* |
|  | 4 | 0.65-0.7 | *qG_3d_4.1* |  |  |  | 3.8E-04 | -0.13 | 2.2 |  |
|  | 4 | 30.04-30.11 | *qG_3d_4.2* |  |  |  | 3.2E-03 | -0.09 | 1.3 |  |
|  | 5 | 24.99-25.3 | *qG_3d_5.1* | 7.0E-04 | -9.76 | 4 | 4.2E-03 | -0.08 | 1.2 | *FHS5.1* |
|  | 5 | 29.0-29.61 | *qG_3d_5.2* | 3.6E-04 | -5.31 | 1.8 | 4.8E-05 | -0.12 | 1.9 |  |
|  | 6 | 0.42-0.48 | *qG_3d_6.1* |  |  |  | 1.4E-03 | -0.14 | 1.5 |  |
|  | 6 | 9.3-9.44 | *qG_3d_6.2* |  |  |  | 5.9E-10 | -0.37 | 9.7 | *Sdr9* |
|  | 6 | 21.07-21.13 | *qG_3d_6.3* |  |  |  | 2.8E-07 | -0.2 | 5.2 |  |
|  | 8 | 3.5-4.14 | *qG_3d_8.1* | 3.0E-03 | -1.61 | 2.3 | 1.3E-03 | -0.13 | 1.5 |  |
|  | 8 | 22.85-25.08 | *qG_3d_8.2* | 8.0E-05 | -3.03 | 8.1 |  |  |  | *FHS8.1* |
|  | 9 | 9.23-9.37 | *qG_3d_9* |  |  |  | 8.5E-05 | -0.13 | 2.8 |  |
|  | 10 | 20.28-20.38 | *qG_3d_10* |  |  |  | 2.3E-04 | -0.13 | 2 |  |
|  | 11 | 18.6-21.0 | *qG_3d_11.1* | 3.6E-05 | -2.81 | 3.2 |  |  |  |  |
|  | 11 | 23.23-23.86 | *qG_3d_11.2* | 1.3E-06 | -4.67 | 9.6 | 1.1E-03 | -0.1 | 3.5 |  |
|  | 11 | 28.09-28.22 | *qG_3d_11.3* |  |  |  | 1.2E-03 | -0.09 | 2.7 |  |
| AUC | 1 | 17.95-17.99 | *qAUC1.1* |  |  |  | 2.0E-03 | 0.04 | 1.6 |  |
|  | 1 | 26.71-26.88 | *qAUC1.2* |  |  |  | 6.8E-04 | 0.08 | 0.3 |  |
|  | 1 | 30.4-33.3 | *qAUC1.3* | 3.7E-04 | 0.88 | 4.7 |  |  |  | *FHS1.1* |
|  | 1 | 35.8-36.1 | *qAUC1.4* | 3.5E-03 | 0.84 | 2.9 |  |  |  |  |
|  | 2 | 7.38-7.41 | *qAUC2* |  |  |  | 9.8E-05 | -0.09 | 2.7 |  |
|  | 3 | 0.05-0.70 | *qAUC3.1* | 3.2E-05 | -1.14 | 6.7 | 2.6E-09 | -0.17 | 6.4 | *OsG* |
|  | 3 | 11.68-11.76 | *qAUC3.2* |  |  |  | 2.6E-03 | 0.14 | 0.5 |  |
|  | 3 | 27.4-28.45 | *qAUC3.3* | 9.2E-09 | -1.53 | 14.2 | 6.8E-09 | -0.13 | 9.5 | *qPHS3* |
|  | 4 | 0.65-0.7 | *qAUC4* |  |  |  | 8.3E-06 | -0.11 | 3 |  |
|  | 5 | 1.07-1.31 | *qAUC5.1* |  |  |  | 4.6E-03 | 0.07 | 0.3 |  |
|  | 5 | 24.99-25.3 | *qAUC5.2* | 3.2E-03 | -2.11 | 2.5 | 3.7E-04 | -0.07 | 2.5 | *FHS5.1* |
|  | 5 | 29.0-29.61 | *qAUC5.3* | 2.6E-03 | -1.21 | 2.2 |  |  |  |  |
|  | 6 | 0.42-0.48 | *qAUC6.1* |  |  |  | 5.9E-05 | -0.12 | 1.7 |  |
|  | 6 | 9.3-9.44 | *qAUC6.2* |  |  |  | 4.1E-09 | -0.23 | 8.3 | *Sdr9* |
|  | 6 | 20.97-21.13 | *qAUC6.3* |  |  |  | 2.4E-05 | -0.11 | 3.9 | *Sdr10* |
|  | 7 | 22.48-22.52 | *qAUC7* |  |  |  | 1.1E-03 | 0.07 | 1 | *Sdr4* |
|  | 8 | 4.07-4.14 | *qAUC8.1* |  |  |  | 3.1E-03 | -0.08 | 1.3 |  |
|  | 8 | 20.41-20.45 | *qAUC8.2* |  |  |  | 2.1E-03 | -0.08 | 1.6 |  |
|  | 8 | 22.85-25.08 | *qAUC8.3* | 1.2E-05 | -0.83 | 7.2 |  |  |  | *FHS8.1* |
|  | 9 | 9.62-9.69 | *qAUC9.1* |  |  |  | 6.0E-04 | -0.08 | 3 |  |
|  | 9 | 20.42-20.48 | *qAUC9.2* |  |  |  | 6.4E-04 | -0.09 | 4 |  |
|  | 10 | 20.28-20.38 | *qAUC10* |  |  |  | 1.4E-04 | -0.09 | 3 |  |
|  | 11 | 18.6-21.0 | *qAUC11.1* | 5.7E-04 | -0.72 | 3.2 |  |  |  |  |
|  | 11 | 23.23-23.86 | *qAUC11.2* | 2.1E-05 | -1.09 | 8.3 | 7.7E-05 | -0.08 | 4.8 |  |
|  | 11 | 28.09-28.22 | *qAUC11.3* |  |  |  | 2.5E-03 | -0.06 | 3.2 |  |
| T_50_ | 1 | 17.95-17.99 | *qT_50_1.1* |  |  |  | 1.0E-03 | -0.06 | 1.5 |  |
|  | 1 | 26.71-26.88 | *qT_50_1.2* |  |  |  | 2.7E-03 | -0.08 | 0.1 |  |
|  | 1 | 30.4-33.3 | *qT_50_1.3* | 4.8E-03 | -1.18 | 4.4 |  |  |  | *FHS1.1* |
|  | 1 | 38.85-38.97 | *qT_50_1.4* |  |  |  | 2.4E-03 | -0.06 | 0.7 | *Sd1* |
|  | 2 | 7.38-7.41 | *qT_50_2.1* |  |  |  | 7.9E-04 | 0.1 | 2.1 |  |
|  | 2 | 12.2-14.3 | *qT_50_2.2* | 3.1E-03 | 2.01 | 5.9 |  |  |  |  |
|  | 3 | 0.05-0.70 | *qT_50_3.1* | 6.5E-05 | 1.93 | 7.6 | 1.3E-08 | 0.19 | 5.9 | *OsG* |
|  | 3 | 14.38-14.5 | *qT_50_3.2* |  |  |  | 1.5E-03 | 0.25 | 2.1 |  |
|  | 3 | 27.4-28.45 | *qT_50_3.3* | 2.8E-08 | 2.54 | 17 | 3.1E-08 | 0.15 | 9 | *qPHS3* |
|  | 4 | 0.65-0.7 | *qT_50_4* |  |  |  | 2.5E-04 | 0.1 | 2.3 |  |
|  | 5 | 1.02-1.07 | *qT_50_5.1* |  |  |  | 8.0E-04 | -0.09 | 0.6 |  |
|  | 5 | 24.99-25.14 | *qT_50_5.2* |  |  |  | 7.9E-04 | 0.08 | 2 | *FHS5.1* |
|  | 5 | 29-29.15 | *qT_50_5.3* |  |  |  | 2.8E-04 | 0.08 | 1.8 |  |
|  | 6 | 0.42-0.48 | *qT_50_6.1* |  |  |  | 5.3E-05 | 0.14 | 2 |  |
|  | 6 | 9.3-9.44 | *qT_50_6.2* |  |  |  | 5.5E-07 | 0.24 | 6.5 | *Sdr9* |
|  | 6 | 20.97-21.13 | *qT_50_6.3* |  |  |  | 7.4E-05 | 0.12 | 3.5 | *Sdr10* |
|  | 7 | 22.48-22.52 | *qT_50_7* |  |  |  | 1.2E-03 | -0.08 | 1.5 | *Sdr4* |
|  | 8 | 4.07-4.14 | *qT_50_8.1* |  |  |  | 3.2E-03 | 0.09 | 1.4 |  |
|  | 8 | 22.85-25.08 | *qT_50_8.2* | 3.0E-03 | 1.06 | 8.2 |  |  |  | *FHS8.1* |
|  | 9 | 9.62-9.69 | *qT_50_9.1* |  |  |  | 7.6E-04 | 0.09 | 3.2 |  |
|  | 9 | 20.39-20.42 | *qT_50_9.2* |  |  |  | 2.6E-03 | 0.09 | 4.3 |  |
|  | 10 | 20.28-20.38 | *qT_50_10* |  |  |  | 1.6E-05 | 0.13 | 3.7 |  |
|  | 11 | 10.97-11.01 | *qT_50_11.1* |  |  |  | 2.2E-03 | -0.06 | 0.2 |  |
|  | 11 | 18.6-21.0 | *qT_50_11.2* | 1.8E-04 | 1.23 | 4.4 |  |  |  |  |
|  | 11 | 23.23-23.86 | *qT_50_11.3* | 4.0E-05 | 1.87 | 7.3 | 7.7E-04 | 0.08 | 4.2 |  |
|  | 11 | 28.09-28.22 | *qT_50_11.4* |  |  |  | 2.3E-03 | 0.07 | 3.3 |  |
| G_7d_ | 1 | 17.95-17.99 | *qG_7d_1.1* |  |  |  | 1.5E-03 | 0.04 | 0.7 |  |
|  | 1 | 26.71-26.88 | *qG_7d_1.2* |  |  |  | 1.1E-03 | 0.06 | 0.4 |  |
|  | 1 | 33.82-33.94 | *qG_7d_1.3* |  |  |  | 1.7E-03 | -0.04 | 1.5 |  |
|  | 3 | 0.05-0.70 | *qG_7d_3.1* | 2.2E-09 | -1.71 | 11.9 | 2.4E-04 | -0.07 | 3 | *OsG* |
|  | 3 | 4.27-4.31 | *qG_7d_3.2* |  |  |  | 2.9E-03 | -0.05 | 1.4 | *OsPDS* |
|  | 3 | 27.4-28.45 | *qG_7d_3.3* | 6.4E-13 | -2.02 | 24.1 | 6.2E-05 | -0.07 | 4.6 | *qPHS3* |
|  | 4 | 0.49-0.7 | *qG_7d_4.1* |  |  |  | 1.8E-05 | -0.09 | 3.6 |  |
|  | 4 | 31.91-32.9 | *qG_7d_4.2* | 2.7E-03 | -0.53 | 4 |  |  |  |  |
|  | 5 | 25.3-27.4 | *qG_7d_5.1* | 2.6E-06 | -3.43 | 8 |  |  |  |  |
|  | 5 | 28.71-29.61 | *qG_7d_5.2* | 6.8E-05 | -1.67 | 2.7 | 7.0E-04 | -0.05 | 2.3 |  |
|  | 6 | 8.96-9.44 | *qG_7d_6.1* |  |  |  | 5.7E-13 | -0.21 | 14.1 | *Sdr9* |
|  | 6 | 20.8-21.13 | *qG_7d_6.2* |  |  |  | 5.0E-13 | -0.15 | 8.3 | *Sdr10* |
|  | 8 | 17.82-18.04 | *qG_7d_8.1* |  |  |  | 1.2E-03 | -0.1 | 1.6 |  |
|  | 8 | 22.85-25.08 | *qG_7d_8.2* | 4.1E-07 | -1.01 | 8.6 |  |  |  | *FHS8.1* |
|  | 9 | 9.44-9.62 | *qG_7d_9* |  |  |  | 2.9E-03 | -0.06 | 2 |  |
|  | 10 | 19.78-19.86 | *qG_7d_10* |  |  |  | 4.5E-03 | -0.06 | 0.6 |  |
|  | 11 | 11.01-11.12 | *qG_7d_11.1* |  |  |  | 3.2E-03 | 0.03 | 0.1 |  |
|  | 11 | 23.24-23.86 | *qG_7d_11.2* | 7.7E-05 | -1.06 | 8.7 |  |  |  |  |

^a^ G_3d_: germination rate at 72 h after imbibition; G_7d_: maximum germination rate at 168 h after imbibition; T_50_: germination speed, which is the time to reach 50% germination of seeds; and AUC: the area under the curve up to 168 h after imbibition. ^b^ Chr: Chromosome. ^c^ QTL: quantitative trait loci. ^d^ PVE (%): phenotypic variance explained by a given QTL. The positive effect for G_3d_, AUC and G_7d_ indicate the NIP alleles increased seed dormancy, and the positive effect for T_50_ represents the NIP alleles decreased seed dormancy. Physical position of a given bin is based on the database MSU7.0 (http://rice.plantbiology.msu.edu/index.shtml).
