# Supplementary material for: Genetic Dissection of Seed Dormancy in Rice (Oryza sativa L.) by Using Two Mapping Populations Derived from Common Parents
Source: Rice (N Y). 2020 Aug 5;13:52. doi: 10.1186/s12284-020-00413-4 (PMC7406625; doi:10.1186/s12284-020-00413-4)
Supplement: Supplementary file 8 — Additional file 8: Table S6. QTLs detected in the same or overlapping regions for seed dormancy in CSSLs and BILs. [file 12284_2020_413_MOESM8_ESM.docx]

**Table S6.** QTLs detected in the same or overlapping for seed dormancy in CSSLs and BILs

| QTL cluster | ^a^Chr | Interval (Mb) | G_3d_ | AUC | T_50_ | G_7d_ | Population |
| --- | --- | --- | --- | --- | --- | --- | --- |
| *qSD1.1* | 1 | 17.95-17.99 | *qG_3d_1.1* | *qAUC1.1* | *qT_50_1.1* | *qG_7d_1.1* | BILs |
| *qSD1.2* | 1 | 26.70-26.88 | *qG_3d_1.2* | *qAUC1.2* | *qT_50_1.2* | *qG_7d_1.2* | BILs |
| *qSD1.3* | 1 | 30.39-33.30 | *qG_3d_1.3* | *qAUC1.3* | *qT_50_1.3* | *-* | CSSLs |
| *qSD2* | 2 | 7.38-7.41 | *qG_3d_2.2* | *qAUC2* | *qT_50_2.1* | *-* | BILs |
| *qSD3.1* | 3 | 0.35-0.59 | *qG_3d_3.1* | *qAUC3.1* | *qT_50_3.1* | *qG_7d_3.1* | BILs |
| *qSD3.1* | 3 | 0.39-0.70 | *qG_3d_3.1* | *qAUC3.1* | *qT_50_3.1* | *qG_7d_3.1* | CSSLs |
| *qSD3.2* | 3 | 27.99-28.26 | *qG_3d_3.3* | *qAUC3.3* | *qT_50_3.3* | *qG_7d_3.3* | BILs |
| *qSD3.2* | 3 | 27.4-28.45 | *qG_3d_3.3* | *qAUC3.3* | *qT_50_3.3* | *qG_7d_3.3* | CSSLs |
| *qSD4* | 4 | 0.65-0.69 | *qG_3d_4.1* | *qAUC4* | *qT_50_4* | *qG_7d_4.1* | BILs |
| *qSD5.1* | 5 | 24.99-25.3 | *qG_3d_5.1* | *qAUC5.2* | *qT_50_5.2* | *-* | BILs |
| *qSD5.2* | 5 | 28.71-29.61 | *qG_3d_5.2* | *qAUC5.3* | *-* | *qG_7d_5.2* | CSSLs |
| *qSD5.2* | 5 | 29.00-29.14 | *qG_3d_5.2* | - | *qT_50_5.3* | *qG_7d_5.2* | BILs |
| *qSD6.1* | 6 | 0.42-0.48 | *qG_3d_6.1* | *qAUC6.1* | *qT_50_6.1* | *-* | BILs |
| *qSD6.2* | 6 | 9.30-9.43 | *qG_3d_6.2* | *qAUC6.2* | *qT_50_6.2* | *qG_7d_6.1* | BILs |
| *qSD6.3* | 6 | 20.8-20.96 | *qG_3d_6.3* | *qAUC6.3* | *qT_50_6.3* | *qG_7d_6.2* | BILs |
| *qSD8.1* | 8 | 4.06-4.13 | *qG_3d_8.1* | *qAUC8.1* | *qT_50_8.1* | *-* | BILs |
| *qSD8.2* | 8 | 22.85-25.08 | *qG_3d_8.2* | *qAUC8.3* | *qT_50_8.2* | *qG_7d_8.2* | CSSLs |
| *qSD9* | 9 | 9.23-9.37 | *qG_3d_9* | *qAUC9.1* | *qT_50_9.1* | *qG_7d_9* | BILs |
| *qSD10* | 10 | 20.28-20.38 | *qG_3d_10* | *qAUC10* | *qT_50_10* | *-* | BILs |
| *qSD11.1* | 11 | 18.6-21.0 | *qG_3d_11.1* | *qAUC11.1* | *qT_50_11.2* | *-* | CSSLs |
| *qSD11.2* | 11 | 23.23-23.26 | *qG_3d_11.2* | *qAUC11.2* | *qT_50_11.3* | *-* | BILs |
| *qSD11.2* | 11 | 23.24-23.86 | *qG_3d_11.2* | *qAUC11.2* | *qT_50_11.3* | *qG_7d_11.2* | CSSLs |
| *qSD11.3* | 11 | 28.09-28.22 | *qG_3d_11.3* | *qAUC11.3* | *qT_50_11.4* | *-* | BILs |

^a^ Chr: Chromosome; G_3d_: germination rate at 72 h after imbibition; G_7d_: maximum germination rate at 168 h after imbibition; T_50_: germination speed, which is the time to reach 50% germination of seeds; and AUC: the area under the curve up to 168 h after imbibition; CSSLs: chromosome segment substitution lines; BILs: backcross inbred lines. “–” indicates data not available.
