# Supplementary material for: Genetic Dissection of Seed Dormancy in Rice (Oryza sativa L.) by Using Two Mapping Populations Derived from Common Parents
Source: Rice (N Y). 2020 Aug 5;13:52. doi: 10.1186/s12284-020-00413-4 (PMC7406625; doi:10.1186/s12284-020-00413-4)
Supplement: Supplementary file 9 — Additional file 9: Table S7. Epistatic interaction of qSD3.1 and qSD3.2 in the NY61-derived population. [file 12284_2020_413_MOESM9_ESM.docx]

**Table S7.** Epistatic interaction of *qSD3.1* and *qSD3.2* in the NY61-derived population

| QTL | DF | F | *P* value |
| --- | --- | --- | --- |
| *qSD3.1* | 2 | 123.4 | 0.0000 |
| *qSD3.2* | 2 | 88.7 | 2.9 × 10^-24^ |
| *qSD3.1 × qSD3.2* | 4 | 2.9 | 0.023 |
